# The Evaluation of Serum KIM-1 in a Pediatric Cohort of Renal Transplantation—A Pilot Study

**DOI:** 10.3390/children12010063

**Published:** 2025-01-07

**Authors:** Paul Luchian Aldea, Roxana Andreea Turbuleasa-Jurje, Bogdan Bulata, Dan Delean, Florin Ioan Elec, Lorena Ciumarnean, Andreea Liana Bot (Rachisan)

**Affiliations:** 1 Renal Transplantation Unit, Urology Department, University of Medicine and Pharmacy “Iuliu Hatieganu”, 400023 Cluj-Napoca, Romania; luchian97@yahoo.com (P.L.A.);; 2 Department of Pediatric Nephrology, Cluj-Napoca Children’s Hospital Gheorghieni, 400023 Cluj-Napoca, Romania; roxana.jurje@yahoo.com (R.A.T.-J.); bogdan.bulata@yahoo.com (B.B.);; 3 Faculty of Nursing and Health Sciences, Department 2, University of Medicine and Pharmacy “Iuliu Hatieganu”, 400023 Cluj-Napoca, Romania; lorena.ciumarnean@umfcluj.ro

**Keywords:** kidney transplantation, KIM-1, biomarkers, graft dysfunction

## Abstract

Introduction: Renal transplantation ensures particular advantages for patients with end-stage kidney disease. However, in some cases, early complications may result in allograft dysfunction, which can ultimately lead to the loss of the graft. Creatinine is a poor biomarker for kidney injury due principally to its inability to help diagnose early acute renal failure and complete inability to help differentiate among its various causes. Different urinary and serum proteins have been intensively investigated as possible biomarkers in this setting. We focused on emerging serum biomarkers such as kidney injury molecule 1 (KIM-1) on a cohort of grafted patients. The motivation of this study was to analyze a predictive biological marker in comparison with standard markers for the evaluation of renal function, with the aim of observing if there are statistically significant differences regarding the performance and promptness of its increase compared to the current monitoring methods in order to improve graft survival, quality of life, and overall patient prognosis. Patients and Methods: We included 21 patients who had their first kidney transplantation (8 females, 13 males), with a follow-up period from transplantation of 3.14 years, without prior immunization, having complete HLA typing and a negative cross-match test before transplantation. We determined serum creatinine and KIM-1 in the whole cohort at the time of the enrollment in the study. Results: The mean creatinine value was 0.89 mg/dL ± 0.33. The mean value for KIM-1 was 13.56 +/− 21.52 in the Tx group vs. 5.91 +/− 3.26 in the control group with a *p*-value of 0.06. We defined patients at low risk (LR) of graft loss (serum creatinine < 0.9 mg/dL) and those at high risk (HR) (serum creatinine > 0.91 mg/dL). The mean values for KIM-1 were 6.09 +/− 1.67 in the LR vs. 21.77 +/− 29.71 in the HR group, with a *p*-value 0.01. Conclusions: There is a strong difference for KIM-1 at 24 h postTx between the two groups, showing a high correlation between KIM-1 and the predisposition of the graft dysfunction. Further studies are needed in order to clarify the utility of these novel biomarkers in the prediction of graft survival in renal transplantation patients.

## 1. Introduction

Currently, kidney transplantation is the most appropriate treatment for end-stage chronic kidney disease. Early detection of the risk of post-transplant graft dysfunction is very important for renal graft survival and overall patient prognosis. The most commonly used standard markers of renal function, serum creatinine and serum urea, fail to detect this decline in renal function early enough; thus, there is a need for new, high-performance markers to assess this risk. Kidney injury molecule 1 (KIM-1) or TIM-1 is a type I transmembrane glycoprotein expressed in high amounts at the apical membrane of proximal convoluted tubule cells following renal injury of various causes (acute kidney injury, chronic kidney disease, acute/chronic graft rejection, nephrotoxic medication, ischemia of other etiologies) and also in lymphocytes and is involved in modulating immune responses [1]. KIM-1 activity is correlated with physiological, adaptive, and pathophysiological processes (e.g., internalization of certain virion types into the cell, progression of fibrosis due to long-lasting expression in renal epithelial cells). It has two extracellular domains (immunoglobulin-like and mucin-like), a transmembrane domain, and an intracellular domain [2]. The use of KIM-1 presents a number of advantages: it is secreted mainly in the kidney, its physiologic level is extremely low, its expression increases rapidly after the onset of acute tubular necrosis (within a few hours and visibly within the first 24 h), and its amount correlates with the severity of acute and chronic renal injury. KIM-1 is detectable in both acute and chronic renal injury and has both a diagnostic and prognostic role, with a higher sensitivity and specificity than creatinine [3,4]. Due to its immunoglobulin-like extracellular domain, KIM-1 in renal epithelial cells functions as a receptor that, by binding phosphatidylserine, has the ability to recognize apoptotic cells and redirect them to lysosomes, thus playing a role in phagocytosis. Thus, it leads to the elimination of cellular debris resulting from renal injury of various etiologies and participates in the modulation of the immune response in AKI (decrease in pro-inflammatory response). Through this metalloproteinase-mediated process, the extracellular domain is released into the tubular lumen and becomes detectable in urine as well as in blood [2]. KIM-1 has both a role as a biomarker of kidney injury and as a protective agent (at least in the initial phases of acute kidney injury) through cell regeneration and repair. Maintenance of elevated KIM-1 levels, however, contributes to the maintenance of the pro-inflammatory status, resulting in progression to renal fibrosis and tubular apoptosis [5,6]. With regard to early detection of nephrotoxicity by certain therapeutic agents, there are many studies supporting KIM-1 for signaling cisplatin-induced acute kidney injury, increasing significantly before creatinine. Favorable results have also been reported in animal studies for the detection of nephrotoxicity induced by gentamicin and cyclosporine [7]. As for its determination post renal transplantation, it has been observed that a high KIM-1 level in the first 24 h correlates with an increased risk of acute rejection. In addition, it also appears to have a promising role in the detection of chronic rejection, and future studies are needed [6].

The motivation of this study is to analyze a predictive biomarker in comparison with standard markers of renal function assessment in order to observe whether there are statistically significant differences in their performance and timeliness of increase compared to current monitoring methods to ultimately lead to improved graft survival, quality of life, and overall patient prognosis.

## 2. Materials and Methods

### 2.1. Study Population

A retrospective cohort study was developed, in which 21 pediatric patients who underwent renal transplantation between January 2011 and August 2023 were consecutively enrolled. Patients were selected after prior HLA typing after performing a negative crossmatch (between donor and recipient), and 90.47% (19 patients) of patients received on the day of transplantation a standard triple therapy (tacrolimus and mycophenolate inhibitor, to which was added induction therapy with anti IL2 receptor antibodies) and 2 patients received cyclosporine A instead of tacrolimus. In addition, a control group of 13 non-transplanted individuals with normal renal function were included in the study.

### 2.2. Sample Collection and Biomarker Assays

All the serum samples were collected at the time of the patients’ enrollment in the study, over a period of 3 months, starting June 2023 until August 2023. The assessment of kidney function was based on evaluating the serum creatinine and KIM-1 concentration at this time point. The mean interval period from renal transplantation to study enrollment was 3.14 years. Serum samples were collected during the first morning hours, patients being a jeun. Following that, serum specimens were centrifugated at 3500 revolutions per minute for 5 min and stored at −20 °C. Samples stored for more than one month were kept at −80 °C.

Serum KIM-1 levels were measured in duplicate using a microbead-based assay as previously described [8]. The samples were diluted 1:10, in a diluent buffer. Thirty microliters (30 μL) of diluted sample, recombinant standards, and internal control samples were incubated with microbeads coupled with KIM-1 capture antibody for 1 h. Beads were then washed 3× with PBST and incubated with detection antibody for 45 min. Beads were washed 3× with PBS-Tween and incubated with Streptavidin-PE (Invitrogen, UK) for 15 min. The signal from the fluorochrome, which is directly proportional to the amount of antigen bound at the micro-bead surface, was captured using Bio-Plex 200 system (Bio-Rad). Data were generated and interpreted using a five parametric logistic regression analysis.

### 2.3. Statistical Analysis

All obtained data were compiled into a database created using Microsoft Office Excel Version 16.78.3 and subsequently analyzed with IBM SPSS Statistics Version 20. For the continuously distributed variables, we applied descriptive statistics to calculate the averages and standard deviations. To assess the statistical relevance of these variables, we utilized Student’s *t*-test. To assess the statistical significance of the correlations between the qualitative variables, we utilized the Pearson Chi-square test. Additionally, we calculated the Pearson correlation coefficient (Pearson r) and created graphical representations of the regression line to illustrate these correlations. Across all analyses, a *p*-value of less than 0.05 was deemed statistically significant. For *p*-values less than 0.01, we considered the statistical significance to be good, and for *p*-values less than 0.001, the statistical significance was regarded as extremely important, indicating a very high level of confidence in the results with an error margin of just 0.1%.

## 3. Results

### 3.1. Descriptive Statistics and Creatinine Analyze in the Whole Cohort

The study cohort included 21 pediatric patients undergoing first kidney transplantation. Kidney grafts were taken from both living and deceased donors. Transplantation was either preemptive or after dialysis (hemodialysis (HD)/peritoneal dialysis (PD)). The characteristics and exact values of the cohort parameters are presented in Table 1.

**Figure 1:**
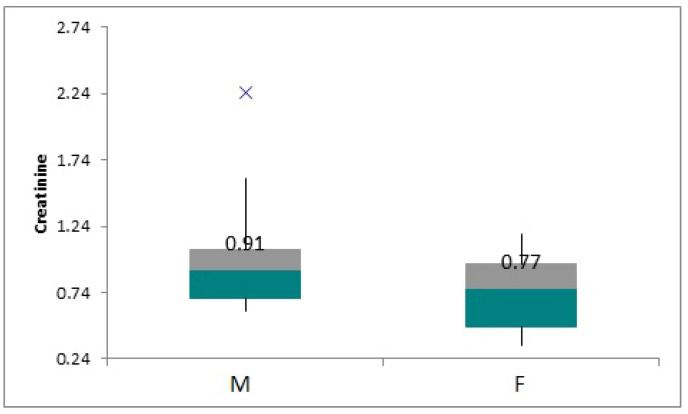
Box-plot showing mean serum creatinine values between sexes.

The patients’ underlying diseases fall into seven categories: hemolytic uremic syndrome (HUS) 9%, Fanconi syndrome 5%, vasculitis 5%, interstitial tubular necrosis (ITN) 5%, uropathy (UPM) 43%, focal segmental glomerulosclerosis (FSGS), and unspecified. Mean serum creatinine value in the entire cohort was 0.89 mg/dL +/− 0.33. The mean serum creatinine value in the male cohort was 0.99 mg/dL +/− 0.19, and in the female cohort it was 0.74 mg/dL +/− 0.09, *p*-value 0.07. The mean serum creatinine values in males (M) and females (F) are shown in Figure 1.

**Figure 2:**
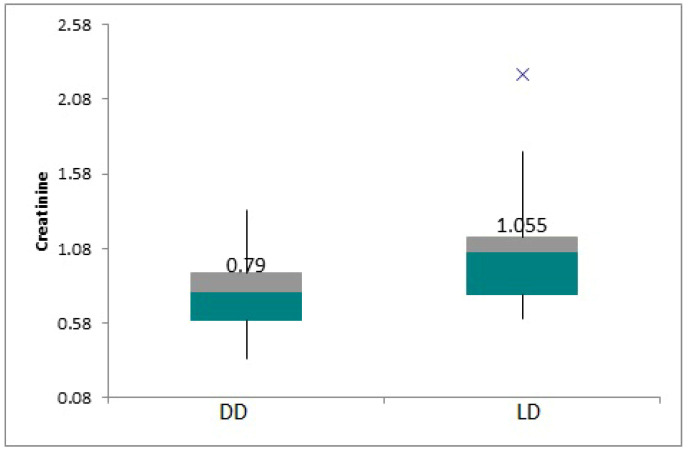
Boxplot showing mean creatinine values by donor type.

For analyzing the mean serum creatinine values according to the background diseases, three broad categories were established: uropathy (UPM), focal segmental glomerulosclerosis (FSGS), and others. The mean creatinine value in the UPM group was 0.94 +/− 0.03, in the FSGS it was 0.85 +/− 0.08, and it was 0.86 +/− 0.43 for the other etiologies. The mean serum creatinine value in the deceased donor (DD) subgroup was 0.77 mg/dL +/− 0.07, and for the living donor subgroup (LD) it was 1.1 mg/dL +/− 0.26, *p* = 0.06. The means for the two subgroups are shown in Figure 2. The serum creatinine value according to the type of epuration was 1.02 +/− 0.39 in hemodialysis patients vs. 0.86 +/− 0.05 in peritoneal dialysis, with a *p* > 0.05, while for the patients having a preemptive graft the mean value was 0.72 +/− 0.04.

### 3.2. KIM-1 Analyzed in the Whole Cohort and Assessment of the Group Risk

**Figure 3:**
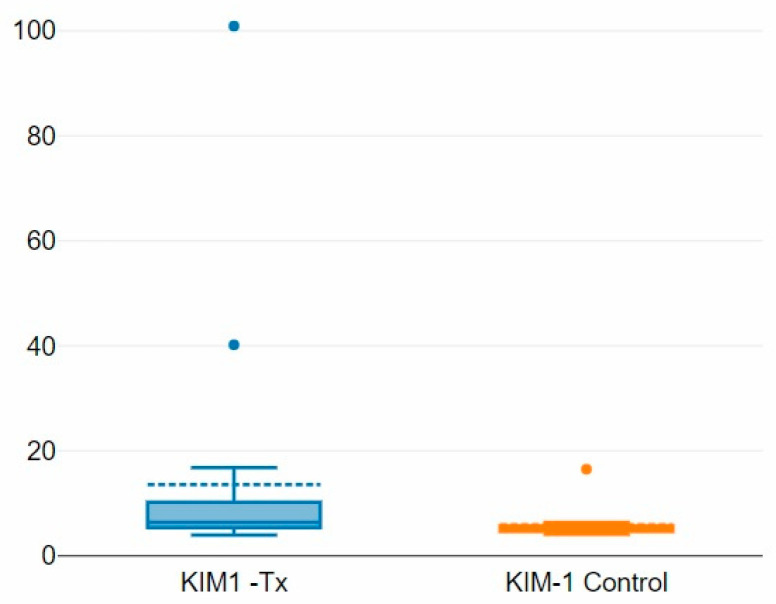
Boxplot showing mean KIM-1 values in the transplanted vs. control group.

**Figure 4:**
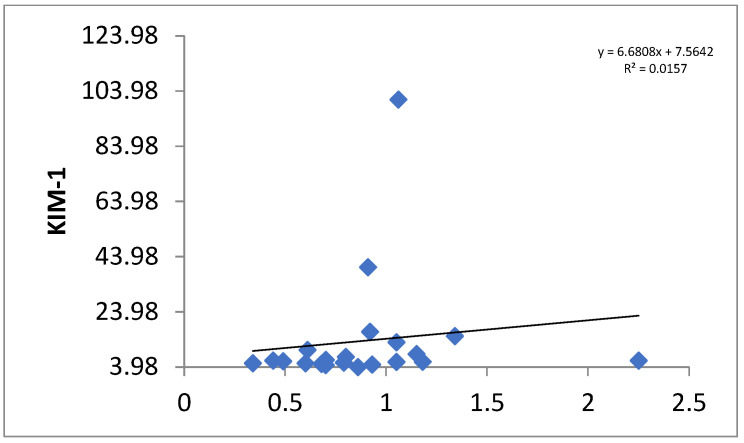
Scatter plot showing the correlation between serum creatinine and KIM-1.

In the transplanted group, the mean KIM-1 value was 13.56 +/− 21.52 and that in the control group was 5.91 +/− 3.26 with a *p*-value of 0.06 (Figure 3). The correlation between the serum creatinine and KIM-1 values for the transplant group is shown in Figure 4.

**Figure 5:**
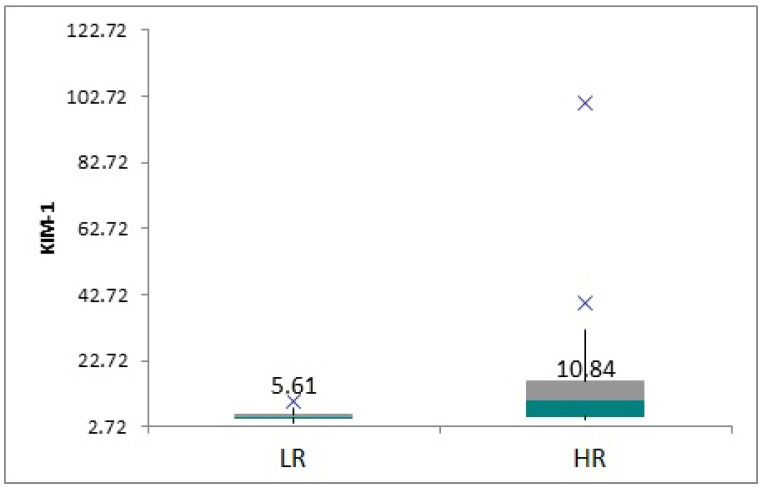
Boxplot showing KIM-1 means in low-risk group vs. high-risk group.

We defined patients at low risk (LR) of graft loss (serum creatinine < 0.9 mg/dL) and those at high risk (HR) (serum creatinine > 0.91 mg/dL). The characteristics of the patients in the two subgroups are shown in Table 2, Figure 5.

## 4. Discussion

End-stage renal disease (ESRD) is diagnosed when kidney function is no longer adequate for long-term survival without kidney transplantation or dialysis [9]. Kidney transplantation is the treatment of choice for pediatric patients with end-stage kidney disease or severe chronic kidney disease as it improves the quality of life and has better survival advantages compared to dialysis. Special considerations must be taken when transplanting children based on the underlying etiology of kidney disease, previous surgical procedures, anatomical limitations, and necessary technical adjustments. Additionally, the choice of donor must be measured to ensure optimal graft survival given a longer post-transplant life expectancy [10]. Advances in immunosuppression and medical care over the past years resulted in better short- and long-term graft survival following kidney transplantation. Novel potent immunosuppressive agents, combinations of proven substances, and the steadily expanding knowledge on the pathophysiology of kidney transplant rejection allow the extension of donor and recipient criteria, including the usage of organs from ABO-incompatible and crossmatch-positive donors, to overcome the increasing problem of organ shortage [11]. Traditional biomarkers such as creatinine remain widely used for assessing kidney function. While creatinine is inexpensive and readily available, it lacks specificity and sensitivity, often identifying injury only at advanced stages. This limitation is particularly evident in children receiving large grafts, where subclinical rejection can occur without detectable changes in serum creatinine, as confirmed by protocol biopsies [12]. In our study, patients were stratified into high-risk and low-risk groups based on serum creatinine levels to investigate their correlation with serum KIM-1 levels. A positive correlation was observed, with higher creatinine levels associated with elevated KIM-1. Importantly, none of the patients in either group have developed graft rejection to date. Previous studies [13,14] demonstrated that creatinine levels measured at one month post-transplant are predictive of long-term graft outcomes. However, our study was limited by the absence of longitudinal evaluation, which would provide more comprehensive insights into dynamic changes over time. KIM-1 has emerged as a promising biomarker for early detection of graft dysfunction. Studies have shown elevated urinary KIM-1 levels in transplant recipients with graft loss [15], while Zhang et al. [16] reported that tissue KIM-1 expression outperforms histology in detecting early tubular injury and correlates with renal dysfunction severity. Increased tissue KIM-1 expression is also predictive of better outcomes and may serve as an early marker of recovery in kidney transplant patients [15]. Furthermore, Nogare et al. [17] evaluated messenger mRNA transcription and gene expression of KIM-1 in kidney tissue and urinary sediment cells from transplant patients with graft dysfunction. They found a significant correlation between KIM-1 gene expression in urine and tissue samples (*p* < 0.01), reinforcing the potential of urinary KIM-1 as a non-invasive surrogate for tissue-based evaluations. Elevated KIM-1 expression was also observed in biopsies with interstitial fibrosis and tubular atrophy, linking it to chronic graft injury [18]. Han et al. [19] demonstrated higher urinary KIM-1 levels in ischemic acute kidney injury compared to non-ischemic forms or chronic kidney disease, highlighting its specificity for ischemic tubular damage, a critical factor in transplant settings where ischemia-reperfusion injury is common. However, Schroppel et al. [20] noted that KIM-1 levels do not reliably predict delayed graft function, underscoring its limitations in specific contexts. KIM-1’s independent association with long-term graft loss [21,22] and its dual role as a marker for both acute kidney injury (AKI) and chronic kidney disease (CKD) [23] emphasize the need for careful interpretation of its levels in diverse clinical scenarios. In addition to KIM-1, other biomarkers have been investigated for their potential in assessing kidney graft function and injury. Neutrophil gelatinase-associated lipocalin (NGAL) is another urinary biomarker with utility in detecting acute tubular injury, particularly in ischemic settings. Elevated NGAL levels have been linked to early graft dysfunction and may complement KIM-1 in identifying tubular damage [24]. Similarly, Liver-type fatty acid-binding protein (L-FABP) has been shown to correlate with renal stress and tubulointerstitial injury, offering another avenue for non-invasive monitoring [25]. N-acetyl-beta-D-glucosaminidase (NAG) is a urinary enzyme that reflects proximal tubular damage and has demonstrated prognostic potential in transplant recipients [26]. These markers, alongside KIM-1, provide a multifaceted approach to monitoring graft health, emphasizing the need for further studies to explore their combined diagnostic value. Despite its potential, the integration of KIM-1 into routine clinical practice remains limited by challenges such as standardization, cost-effectiveness, and validation across diverse populations. However, advances in biomarker detection technologies, including point-of-care devices for KIM-1 measurement, could revolutionize post-transplant monitoring. These tools enable frequent, non-invasive assessment of graft health, reducing reliance on biopsies and improving patient compliance [23]. Further research is necessary to standardize measurement protocols and validate KIM-1’s utility in larger, diverse cohorts, paving the way for its adoption in routine clinical practice.

## 5. Conclusions

We are aware of the limitations of our study. Firstly, the relatively small sample size limits the statistical power of our analyses, and larger studies are needed to validate these results. Secondly, the lack of serial measurements of KIM-1 over time prevents a dynamic evaluation of how these markers fluctuate in response to different stages of recovery or graft dysfunction. Additionally, the effect of immunosuppressive therapy on KIM-1 levels and its correlation with renal function were not explicitly evaluated in our study, which could provide valuable insights into how therapeutic regimens influence biomarker dynamics. Furthermore, our study did not include patients with renal graft rejection, limiting our ability to establish reference values for KIM-1 in this critical subgroup. Despite these limitations, our findings highlight the role of urinary KIM-1 as a promising marker of kidney injury even after successful kidney transplantation. Future studies should focus on larger cohorts, incorporate longitudinal measurements, and explore the interplay between therapy regimens and biomarker profiles. Investigating KIM-1 in diverse patient populations, including those experiencing graft rejections, and across varying clinical scenarios will further clarify its diagnostic and prognostic utility. In conclusion, KIM-1 shows significant potential as a tool for early detection of kidney injury and warrants further investigation to establish its role in the routine monitoring of transplant patients.

## Figures and Tables

**Table 1:** Cohort characteristics (n = 21).

Sex (F/M)	8/13
Mean age at transplantation (years)	11.36 +/− 4.2
No. brain dead donors/No. living donors	13/8
Type of renal clearance (PD/HD)	10/7
Pre-emptive transplantation	4 (19.04%)

**Table 2:** Characteristics of the low- vs. high-risk groups.

	Low Risk (LR) (n = 11)	High Risk (HR) (n = 10)	*p*
Sex (F/M)	5/6	3/7	NS
Preemptive Tx	2	2	NS
DD/LD	8/3	5/5	NS
Mean creatinine ± SD	0.63 +/− 0.12	1.18 +/− 0.39	0.001
Mean KIM-1 ± DS	6.09 +/− 1.67	21.77 +/− 29.71	0.01

## Data Availability

Data is contained within the article.
